# Effects of Leisure Activities on the Cognitive Ability of Older Adults: A Latent Variable Growth Model Analysis

**DOI:** 10.3389/fpsyg.2022.838878

**Published:** 2022-04-13

**Authors:** Chang-e Zhu, Lulin Zhou, Xinjie Zhang

**Affiliations:** Department of Management, Jiangsu University, Zhenjiang City, China

**Keywords:** cognitive ability, leisure activities, development trajectory, latent variable growth model (LGCM), cross-lagged regression analysis

## Abstract

Based on the data of four periods of CLHLS (2008, 2011, 2014, 2018), the latent variable growth model (LGCM) was applied to 2344 older adults who completed four follow-up surveys, to study the trajectory of leisure activities and cognitive ability and explore the relationship between leisure activities and cognitive ability of older adults. The results showed that: (1) leisure activities and cognitive ability of older adults showed a non-linear downward trend; (2) leisure activities significantly and positively predicted the cognitive ability of older adults at every time point; (3) the initial level of leisure activity positively predicted the initial level of cognitive ability but negatively predicted the rate of cognitive decline; In addition, cognitive activities had a greater effect on cognitive ability than non-exercise physical activities; (4) the rate of decline of leisure activities also significantly and positively predicted the rate of decline of cognitive ability; (5) cross-lagged regression analysis further suggested the overall positive predictive effect of leisure activity on cognitive ability; (6) overall, education level had a significant contribution to cognitive ability, and the higher the education level, the slower the decline of cognitive ability; and (7) smoking could promote cognitive ability in older adults and no significant effect was found between alcohol drinking and cognitive ability. Accordingly, the government should encourage older adults to do more leisure activities, especially the cognitive activity, to effectively prevent cognitive decline.

## Background

With the deepening process of population aging, how to promote healthy aging and maintain the physical and mental health of older adults had become the focus of attention from all walks of life. The results of China’s seventh population census revealed that in 2021, China’s older population had reached 264.02 million, accounting for 18.7% of the total population (National Bureau of Statistics of China, 2021). According to the United Nations estimate, by 2050, China’s older population would reach 488 million, accounting for 35.6% of the total population (United Nations, 2012), which meant that China had stepped into a deep aging society, and suffered the problem brought from aging.

Although life expectancy continued to increase, it was necessary to consider adverse outcomes in advance given the increasing likelihood that cognitive ability will decline with age. A previous study showed that deterioration of cognitive ability could lead to physical dysfunction and certain chronic diseases, and severe deterioration of cognitive ability was highly associated with mortality (Yue et al., 2021). Furthermore, the decline of subjective (slight) cognitive ability could be regarded as a precursor of dementia, which seriously impaired the health-related quality of life of older adults (Pusswald et al., 2015).

It was estimated that the number of dementia patients aged 60 and above in China was 10 million to 11 million, the number of dementia patients aged 65 and above was 9 to 10 million, and more than 60% of dementia patients had Alzheimer’s disease (Jia et al., 2020). Accordingly, studying the change trajectory of cognitive ability and exploring the determinants of cognitive ability in older adults, and formulating appropriate intervention policies should be one of the most important tasks for China to address the challenges of population aging. Indeed, previous studies suggested that individuals with more years of education showed less cognitive decline (Liu and Lachman, 2020). But the years of education that older adults received at a young age could not be changed, so it made more sense to study variables that could be modifiable. Studies indicated that leisure activities, as one of the main lifestyles of the elderly in their later years, had an important impact on their cognitive ability. However, the current research conclusions were inconsistent. To be specific, intellectual leisure activities such as reading books and newspapers had a positive impact on cognitive ability (Silverstein and Parker, 2002). However, watching TV was examined to exert an adverse effect on cognitive ability (Rundek and Bennett, 2006; Hamer and Stamatakis, 2014). Accordingly, it was necessary to study the relationship between leisure activities and classify leisure activities, and explain the differences in the impact of different types of leisure activities on cognitive ability.

### Development of Cognitive Ability of Older Adults

Cognitive ability referred to a series of intelligent processing processes in which the human brain received external information and acquires knowledge, which included perception, attention, memory, thinking and other abilities (Ruining and Yinge, 2015). Maintaining the cognitive ability of the older adults was of great significance for the old population in their later life. Firstly, degradation of cognitive ability was strongly associated with morbidity and mortality from a variety of diseases. Secondly, consequences of cognitive decline in older adults not only damaged physical health, but also significantly increased the probability of Alzheimer’s disease (Prince et al., 2013). Last but not least, deterioration of cognitive ability could lead to physical dysfunction and certain chronic diseases, and severe deterioration of cognitive ability was highly associated with mortality (Yue et al., 2021). Therefore, it was important and urgent to study the changing trajectory of cognitive ability of older adults. The theory of human cognitive aging proposed that individual cognition ability would change with age. To be specific, with the increase of age, the neural substrate of people will change, and cognitive resources such as processing speed, working memory, attention span, and inhibitory ability may decline (Wingfield and Grossman, 2006). In other words, the cognitive ability of older adults would decline overtime.

Based on the existing study, the following assumption was proposed:

**H1:** With the increase of age, the cognitive ability of older adults showed a downward trend;

### Development of Leisure Activities for Older Adults

Leisure activity was a kind of meaningful activity which was not related to earning a living or getting a reward. It is an inclusive intellectual activity, multi-dimensional concepts of social, recreational and sports activities (Wuzhen, 2008). The older adults retired from work, so leisure activities became an important arrangement for the older people in their later years. The young elderly (62–69) gradually steps into the ranks of the middle-aged (70–76) and even older adults (77–94) over time (Williams et al., 2012). During this period, the physical function and psychological course of older adults will change, which will further lead to changes in the way of leisure activities. For example, when they were in the low level of aging, outdoor physical exercise and other leisure activities that required much physical exertion were more common than those for middle and older aged people, while older aged people mainly read newspapers and watched TV indoors due to the limitation of their physical conditions and the change of their mental state (Robinson et al., 2004). In addition, leisure activities related to social and intellectual activities also tended to decline due to the lack of energy of older adults. In general, older adults experienced a decline in physical and mental functioning over time, resulting in lower levels of leisure activity. In other words, the level of leisure activities of older people may decrease over time.

Based on the existing literature, the following assumption was proposed:

**H2:** With the increase of age, the leisure activities of older adults showed a downward trend;

### Relationship Between Leisure Activities and Cognitive Ability

At present, there were a large number of studies to analyze the relationship between leisure activities and cognitive ability and leisure activities were considered to be a protective factor for cognitive abilities in older adult (Mao et al., 2020), which would reduce the risk of cognitive decline or dementia (Bennett et al., 2014). For example, a prospective cohort study of community-dwelling adults over 70 years of age found that leisure hobbies significantly reduced the risk of cognitive decline in older adults (Iwasa et al., 2012). In addition, regular outdoor exercise was considered to be an important protection strategy to effectively prevent the decline of cognitive ability (Ding et al., 2021). The theory of social communication held that social activities are an important way for individual human beings to survive, live and practice, a prerequisite for material production and a direct link with individual mental health (Weixiong, 2004). Social interaction can not only stimulate the individual’s thinking, promote older people’s emotional communication, but also improve and promote the older population’s understanding, judgment, memory and expression and other cognitive functions. Previous studies revealed that social activities such as keeping close contact with friends could improve cognitive ability to a certain extent, which was a key factor in effectively preventing cognitive decline (Hui-Xin et al., 2013; Noice et al., 2014). In addition, participation in recreational activities was suggested to be a protective factor against cognitive decline in older adults (Hui-Xin et al., 2002). Finally, numerous studies suggested watching TV was associated with cognitive decline (Hamer and Stamatakis, 2014; Fancourt and Steptoe, 2019). However, watching TV, as a very common form of leisure in old age, was an important source of information for older people and contributed to their overall cognitive function (Ostlund, 2010). Accordingly, leisure activities could be considered to be positively associated with cognitive ability on the whole.

Based on the above analysis, the following assumptions were put forward:

**H3:** Leisure activities of older adults positively predicted their cognitive ability during the same period;

**H4**: The initial level of leisure activities of older population positively predicted the initial level of cognitive ability;

In addition, leisure activities was confirmed to be a protective factor for the cognitive ability of older adults. Leisure activities may reduce the rate of cognitive decline, which indicated that the higher the level of leisure activities, the slower the decline of their cognitive ability. Therefore, the following assumptions were put forward:

**H5:** The initial level of leisure activities of older adults negatively predicted the change speed of their cognitive ability;

The level of leisure activities and cognitive ability of older adults showed signs of decline to some extent over time. According to the life cycle theory (Haizhong, 2014), from the early stage to the middle age stage, the physical and psychological functions of older adults often showed a slow decline. From the middle age to the advanced age stage, the older people reached the last stage of the life cycle, both physical function and cognitive function declined precipitously. In other words, the change speed of physical function and psychological cognition of older population was consistent. Therefore, the following assumptions were proposed:

**H6:** The change rate of leisure activities of older adults positively predicted the change rate of their cognitive ability;

In addition, many studies suggested the positive relationship between leisure activities and cognition ability, which, however, were mainly based on the cross-sectional data or short follow-up period. Cognitive decline is a continuous and insidious process, so it is necessary to prevent and explore its protective factors in advance (Kåreholt et al., 2011). Additionally, cognitive reserve theory suggested that intellectual activities in midlife will created a stock for cognitive capacity, which will influenced their cognitive ability in later life by improving the ability to compensate for age-related neuronal damage (Costa et al., 2007). In other words, baseline leisure time activities were examined to be a protective factor for late-life cognition (Kåreholt et al., 2011). Most importantly, previous studies have proved the positive relationship between leisure activities on cognitive ability, but endogenous problem could not be ruled out (Andel et al., 2015; Lee et al., 2019). Therefore, cross-lagged regression analysis was used to further determine the temporal order of the relationship between leisure activity level and cognitive ability. To verify that there was a causal relationship between two variables, the key step was to ensure that there was a temporal difference between the two variables. In other words, one variable must occur before the other. Hence, it was necessary to study the influence of current leisure activities on cognitive ability in the later period. Therefore, the assumption was put forward:

**H7:** The leisure activities of older adults in the current period positively predicted cognitive ability in the later period.

## Education Differences in Cognitive Ability

The concept of cognitive reserve theory suggested that innate intelligence or life experience (such as educational or professional achievement) may provide reserves in the form of a set of skills to better cope with the decline of cognitive ability (Stern, 2002). Education contributed to the development of brain structure and neural networks, and to the further development of concept formation, vocabulary expression and cognitive functions such as perception and memory (Konttinen et al., 2016). Education was considered a protective factor for cognitive ability, and studies indicated that older people with higher levels of education had higher cognitive abilities (Hazzouri et al., 2011; Ruining and Yinge, 2015). And older adults with higher education had a slower rate of cognitive decline (Sunmin et al., 2003). Therefore, the following assumption was proposed:

**H8:** The educational level of older adults positively predicted their cognitive ability;

To sum up, it was suggested that studies on cognitive abilities of older people were mainly based on cross-sectional data (Saji et al., 2020; Kashibayashi et al., 2021). Furthermore, the existing research on the effect of leisure activities on cognitive ability was mainly based on one leisure activity and did not distinguish the differences in the impact of different types of leisure activities on cognitive ability. In addition, the existing research did not explain the mechanism of the influence of leisure activities on cognitive ability, but simply verified the positive relationship between the levels of two variables, and lacked discussion on the relationship between the initial level of two variables and change rate. Accordingly, longitudinal multi-time data was used to further enrich the connotation of leisure activities and track the change trajectory of cognitive ability and leisure activities of older adults over time. Additionally, leisure activities were categorized into two parts, which included two major aspects: cognitive activities and non-exercise physical activities to study the effects of different types of leisure activities on cognitive ability. Last but not least, this paper studied the influence mechanism of leisure activity on their cognitive ability and discussed the relationship between the initial level of two variables and change rate.

## Method

### Source of Data

**FIGURE 1:**
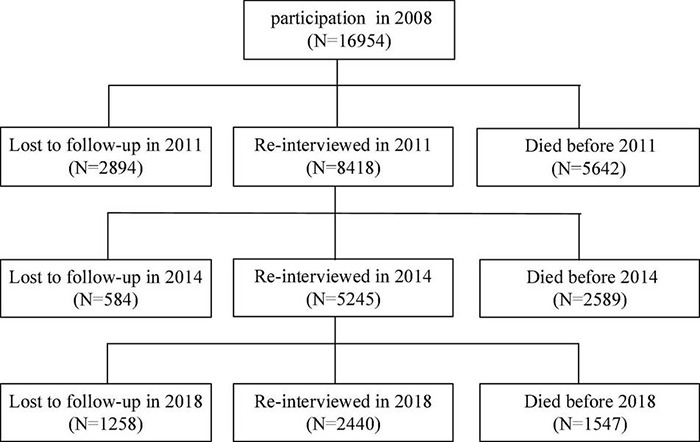
Source of sample structure.

The data were obtained from the Chinese Longitudinal Healthy Longevity Survey (CLHLS), which were conducted by the National Development Institute and the Center for Research on Healthy Aging and development of Peking University. The present study used data from the last four waves of datasets, including the 2008, 2011, 2014, and 2018 waves and the participations in this study were all aged over 60 at the baseline. This paper mainly studied the changing trend of cognitive ability of older adults and the effect of leisure activities on cognitive ability. In order to effectively track the change trajectory of cognitive ability of the older adults, the individuals who simultaneously participated in the survey in 2008, 2011, 2014, and 2018 were included in this study. The sample data acquisition process was shown in Figure 1. The individuals with missing values on any variable were excluded and then a total of 2344 valid individuals were obtained. In addition, for the attrition, the missing completely at random (MCAR) test was conducted to clarify the trend of the missing data and the result was not significant (χ2/df = 6.8, *P* > 0.05), indicating the missing participants were at random.

### Variable Definition and Measurement

The cognitive ability was assessed by Chinese version of the Mini-Mental State Examination (MMSE), which was suggested to be validated in Chinese older population (Zeng et al., 2017; Lv et al., 2018). To be specific, the cognitive ability was measured by general ability (3 items), responsiveness (3 items), attention and calculation ability (6 items), recall (3 items), and language comprehension and self-coordination (6 items), which totally consists of 24 items. Responses were given on dichotomous scale (1 = correct, 0 = wrong) with the exception of the item“number of kinds of food named in 1 min,” which allows a maximum score of 7, so the range for cognitive ability was 0–30. An example item is “What time of day is it right now (morning, afternoon, evening)? The coefficients alpha at every point was 0.863, 0.855, 0.886, and 0.928.

Leisure activities were measured by eight questions, namely (1) housework (cooking and taking care of children), (2) outdoor activities (such as Taijiquan, square dance, crossing the door and communicating with friends), (3) planting flowers and keeping pets, (4) reading books and newspapers, (5) raising poultry and livestock, (6) playing cards or mahjong, (7) listening to the radio, and (8) participating in organized social activities. Responses were given on a five-point Likert scale (1 = almost every day to 5 = rarely or never). An example item is “Do you now perform the following activities regularly (Outdoor activities)?” In this paper, the negative score of the original scale was transformed, so the higher the score was, the higher the level of leisure activities was and the final score range of leisure activities for older adults was 8–40. The coefficients alpha at every point was 0.768, 0.807, 0.856, and 0.908.

A total of 23,44 (1,097 males and 1,247 females) older adults were included in the study (*female* = 0, *male* = 1). Education level was measured by the number of years of education older adults had received (0–22). In addition, lifestyle included whether they had smoked/drunk alcohol was tested (*yes* = 1, *no* = 0). Specifically, 840 (35.8%) people smoked in 2008,837 (35.7%) in 2011, 761 (32.4%) in 2014 and 758 (32.3%) in 2018, 815 (34.8%) drank alcohol in 2008, 821 (35.0%) in 2011, 679 (29.0%) in 2014, and 637 (27.2%) in 2018. The definition of all variables was shown in Table 1.

**TABLE 1:** Descriptive analysis of samples.

	*Definition*	*Mean*	*SD*
*CA1*	The total score of cognitive ability (0–30)	26.77	4.53
*LA 1*	The total score of leisure activity (8–40)	20.95	5.43
*SM1*	Smoked = 1; Never smoked = 0	0.36	0.48
*AD1*	Drunk = 1; never drunk = 0	0.35	0.48
*AGE1*	The true age of older adults (61–108)	75.16	8.29
*EDU*	The years of schooling (0–20)	2.84	3.7
*SEX*	Male = 1; female = 0	0.47	0.5

*CA denotes cognitive ability; CA1 = CA (2008); LA denotes leisure activities; LA1 = LA (2008); SM denotes smoking; SM1 = SM (2008); AD denotes alcohol drinking; AD 1 = AD (2008); EDU denotes the years of schooling; SD denotes standard deviation.*

### Data Analysis Strategy

In this paper, Mplus8.0 was used to construct a latent variable growth model to test the trend of cognitive ability of older people. Latent variable growth model (LGCM) was a variation of structural equation model, which could describe the variation type between repeated measurements by the potential trajectory of the hypothesis. Unlike traditional statistical methods (such as ANOVA with repeated measurements) that focused only on group mean, LGCM could estimate both group and individual variation during development (McArdle, 2008). The LGCM first defined two latent variable structures, i.e., the starting level and the slope. These two latent variable structures were then estimated in the model using the actual measurements of a variable at different time points.

## Results

### Common Method Bias

In this study, the single-factor Harman test was carried out on the data of the four surveys. The results showed that the variation explained by the first factor was less than 40% in the four measurement periods, which were 24.617, 22.647, 26.776, and 34.778%, respectively. This indicated that there was no common method bias in this study (Podsakoff et al., 2003).

### Descriptive Statistical Results

**FIGURE 2:**
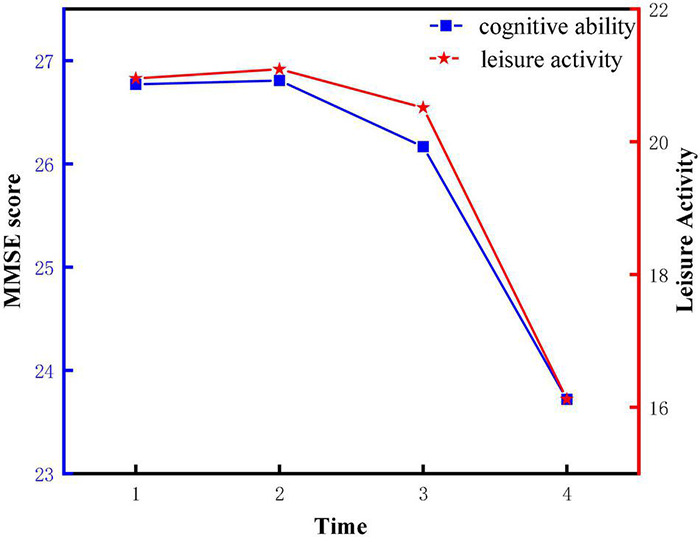
Change trajectory of cognitive ability and leisure activities.

Descriptive statistics and bivariate correlations among all observed variables were shown in Tables 1, 2, respectively. It can be concluded that the average scores for cognitive ability and leisure activity were within 23–27 and 16–22, respectively. Overall, they declined over time. The scores of leisure activities and cognitive abilities of older adults during the four measurement periods were intuitively shown in Figure 2. Additionally, it was suggested that cognitive ability was positively correlated with leisure activities from 2008 to 2018 (T1–T4).

**TABLE 2:** Correlation coefficient matrix.

	*CA1*	*CA 2*	*CA 3*	*CA 4*	*LA 1*	*LA 2*	*LA 3*	*LA 4*	*SM1*	*SM2*	*SM3*	*SM4*	*AD1*	*AD2*	*AD3*	*AD4*
*CA 1*	1															
*CA 2*	0.310***	1														
*CA 3*	0.272***	0.412***	1													
*CA 4*	0.311***	0.383***	0.452***	1												
*LA1*	0.338***	0.225***	0.191***	0.201***	1											
*LA 2*	0.223***	0.308***	0.233***	0.277***	0.310***	1										
*LA 3*	0.246***	0.260***	0.315***	0.354***	0.323***	0.440***	1									
*LA 4*	0.236***	0.269***	0.279***	0.492***	0.294***	0.393***	0.459***	1								
*SM1*	0.101***	0.116***	0.127***	0.144***	0.102***	0.144***	0.130***	0.138***	1							
*SM2*	0.097***	0.118***	0.111***	0.136***	0.105***	0.155***	0.135***	0.137***	0.910***	1						
*SM3*	0.102***	0.108***	0.128***	0.131***	0.074***	0.163***	0.142***	0.130***	0.694***	0.714***	1					
*SM4*	0.103***	0.110***	0.130***	0.127***	0.075***	0.160***	0.151***	0.139***	0.693***	0.695***	0.687***	1				
*AD1*	0.075***	0.070***	0.077***	0.081***	0.098***	0.092***	0.094***	0.087***	0.458***	0.441***	0.370***	0.365***	1			
*AD2*	0.080***	0.071***	0.078***	0.069***	0.086***	0.107***	0.100***	0.080***	0.425***	0.448***	0.379***	0.360***	0.831***	1		
*AD3*	0.087***	0.087***	0.106***	0.116***	0.067***	0.129***	0.153***	0.118***	0.376***	0.394***	0.477***	0.373***	0.569***	0.606***	1	
*AD4*	0.099***	0.091***	0.116***	0.124***	0.072***	0.127***	0.137***	0.133***	0.369***	0.375***	0.377***	0.461***	0.543***	0.559***	0.561***	1

*p ≤ 0.05; **p ≤ 0.01; ***p ≤ 0.001.

### Development Trajectory of Cognitive Ability of Older Adults (Model 1/2)

**FIGURE 3:**
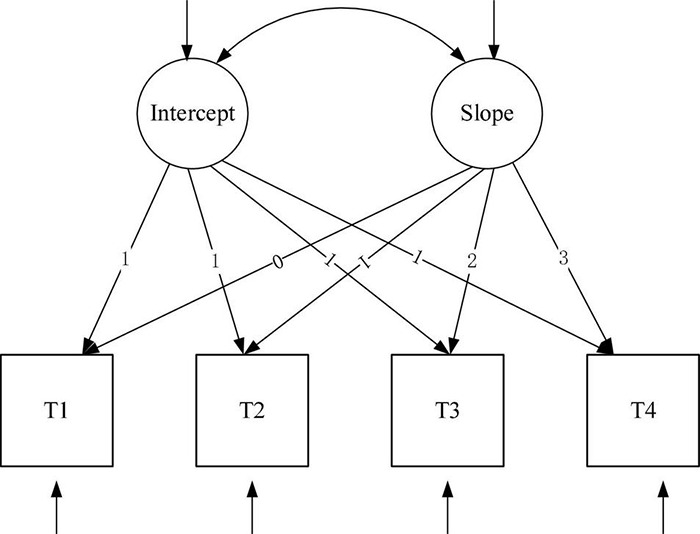
Linear unconditional latent variable growth model.

**FIGURE 4:**
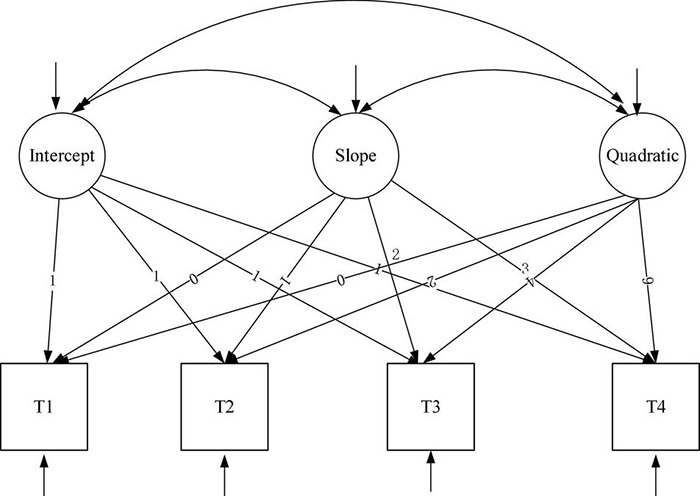
Non-linear unconditional latent variable growth model.

In order to examine the trend of cognitive ability of older people, a linear growth model and quadratic growth model were constructed, which were shown in Figures 3, 4 and denoted as Model 1 (M1) and Model 2 (M2), respectively.

**FIGURE 5:**
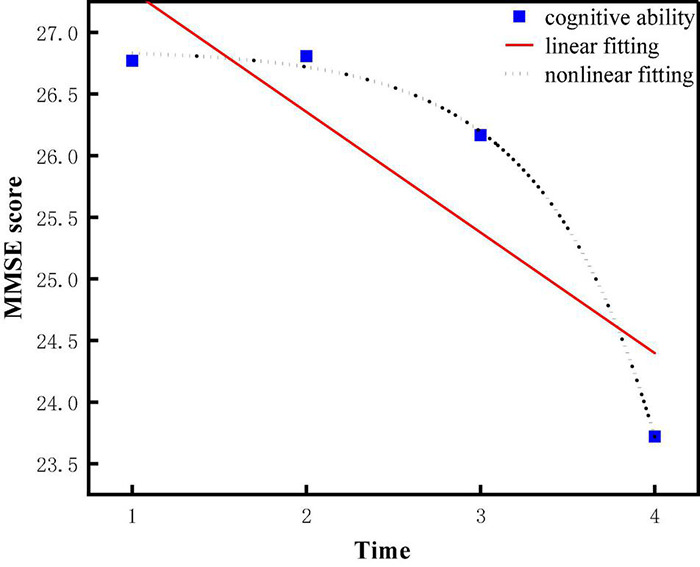
Cognitive ability fitting.

For cognitive ability, both the linear growth model and the quadratic growth model fit the data very well (for the linear growth model 1, χ^2^(df) = 20.7 (5), χ^2^/df = 4.14, CFI = 0.864, RMSEA = 0.131, and SRMR = 0.084, *P* = 0.000; for the quadratic growth model 2, χ^2^(df) = 3.381(1), χ^2^/df = 3.381, CFI = 0.994, RMSEA = 0.063, and SRMR = 0.013, *P* = 0.000). The linear growth model was nested under the quadratic growth model (Chan, 1998), so we compared leisure activities’ linear growth model and quadratic growth model by using the chi-square test. we compared cognitive ability’s linear growth model and quadratic growth model by using the chi-square test. The result was significant (Δχ^2^ = 6.24, Δdf = 2, *P* < 0.05). Furthermore, the fitting effect of quadratic growth model was better than that of linear growth model, which was shown in Figure 5, so we used the quadratic growth model of cognitive ability. To be specific, the initial level of cognitive ability status (the intercept) was 26.729 (*P* < 0.001). Cognitive ability decreased during the four tests (slope = 0.819, *P* < 0.001), and the rate of decline increased year by year (curve slope = −0.593, *P* < 0.01), suggesting a non-linear downward trend in cognitive ability over the four test periods. In addition, the variation of intercept (σ^2^ = 10.108, *P* < 0.001) and slope (σ^2^ = 0.626, *P* < 0.05) were both significantly different among the older population, which indicated that there was significant difference in the initial level and the rate of change of cognitive ability among older adults. Therefore, Hypothesis 1 was supported. Table 3 summarizes the model fit indices of latent growth models for leisure activities and cognitive ability. Table 4 shows parameter estimates of latent growth models for leisure activities and cognitive ability.

**TABLE 3:** Model comparisons for leisure activity and cognitive ability.

Variables	Model	χ^2^(df)	*P*	CFI	RMSEA	SRMR
Cognitive ability	Model 1	20.7(5)	0.000	0.864	0.131	0.084
	Model 2	3.381(1)	0.000	0.994	0.063	0.013,
Leisure activities	Model 3	66.3(5)	0.000	0.584	0.237	0.107
	Model 4	5.69(1)	0.000	0.965	0.054	0.032

**TABLE 4:** Parameter Estimates of latent growth models for leisure activities and cognitive ability.

Variables	Model	Means of growth factors			Variance of growth factors		
		Intercept	Slope	Quadratic	Intercept	Slope	Quadratic
Cognitive ability	Model 1	27.254***	-0.786***		3.799***	0.816***	
	Model 2	26.729***	0.819***	-0.593***	10.108***	8.496***	0.626*
Leisure activities	Model 3	21.976***	-1.587***		8.578***	0.116	
	Model 4	20.773***	1.936***	-1.149**	6.126*	1.836	0.277

### Development Trajectory of Leisure Activities of Older Adults (Model 3/4)

Similarly, in order to examine the trend of leisure activities of the older population, a linear growth model and quadratic growth model were constructed, which were shown in Figures 3, 4 and denoted as Model 3 (M3) and Model 4 (M4), respectively.

For leisure activities, both the linear growth model and the quadratic growth model fit the data very well (for the linear growth model 3, χ^2^ (df) = 66.3 (5), χ^2^/df = 13.26, CFI = 0.584, RMSEA = 0.237, and SRMR = 0.107, *P* = 0.000; for the quadratic growth model 4, χ^2^/(df) = 5.69(1), χ^2^/df = 5.69, CFI = 0.965, RMSEA = 0.054, and SRMR = 0.032, *P* = 0.000). we compared leisure activities’ linear growth model and quadratic growth model by using the chi-square test. The result was significant (Δχ^2^ = 8.23, Δdf = 3, *P* < 0.05).

**FIGURE 6:**
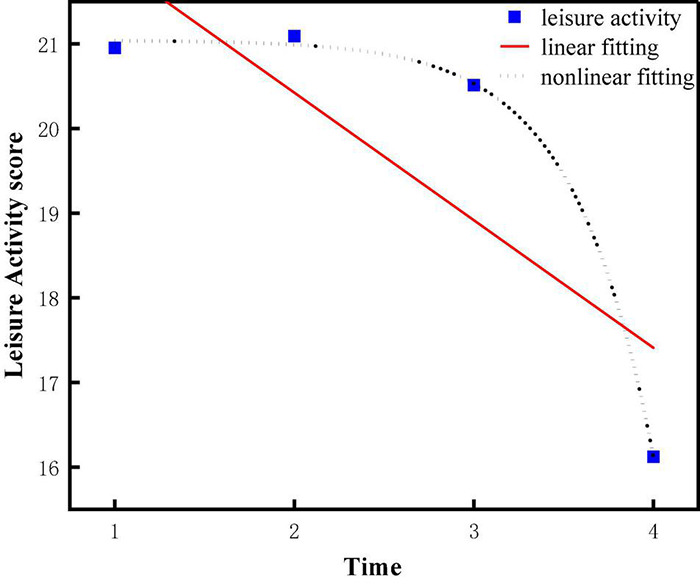
Leisure activity fitting.

In addition, the fitting effect of quadratic growth model was better than that of linear growth model, which could be obviously seen from Figure 6, so we used the quadratic growth model of leisure activities. To be specific, the initial level of leisure activity status (the intercept) was 20.773 (*P* < 0.001). Leisure activities decreased during the four tests (slope = 1.936, *P* < 0.001), and the rate of decline increased year by year (curve slope = −1.149, *P* < 0.001), suggesting a non-linear downward trend in leisure activity over the four test periods. In addition, the variation of intercept was significant (σ^2^ = 6.126, *P* < 0.05) and the variation of curve slope wasn’t significant (σ^2^ = 0.277, *P* > 0.05), which indicated that there were differences in the initial level of leisure activity and no differences in the decline rate of leisure activity level among the older population. Therefore, hypothesis 2 was supported.

### Effects of Leisure Activities on Cognitive Abilities (Model 5)

**FIGURE 7:**
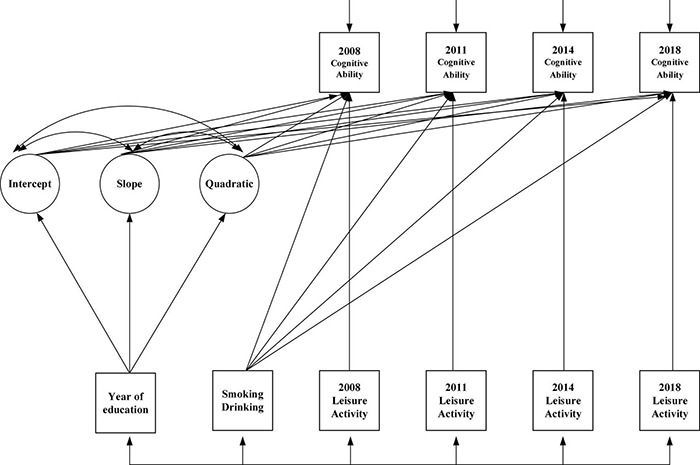
Impact of leisure activities on the change track of cognitive ability.

This study constructed a model with a time-invariant variable (education) and time-variant variables (smoking, alcohol drinking, leisure activities) and treated education, smoking and alcohol drinking as control variables, which were shown in Figure 7. Therefore, this paper mainly studied the effect of leisure activities on cognitive ability.

For model 5, χ^2^*(38)* = 3.2, *P* = 0.000, CFI = 0.929, RMSEA = 0.046, SRMR = 0.055. Education level has a significant effect on initial cognitive ability (γ_0_ = 0.241, *P* < 0.001), which indicated that older adults with high education level have higher cognitive ability. In addition, the value of slope (γ_1_ = −0.071, *P* > 0.05) and curve slope (γ_2_ = 0.027, *P* < 0.05) indicated that the higher the level of education, the slower decline of cognitive ability of the older population. Therefore, Hypothesis 8 was supported.

Importantly, the results showed that at any time point, the more the leisure activity, the higher the cognitive ability (2008: β = 0.218, *P* < 0.001; 2011: β = 0.125, *P* < 0.001; 2014: β = 0.195, *P* < 0.001; 2018: β = 0.499, *P* < 0.001), which meant that leisure activities did promote the cognitive ability of the older people at every time point. Therefore, hypothesis 3 was supported.

In order to examine the effect of different types of leisure activities on cognitive ability, leisure activities were categorized into two types according to the predominant element of each activity (Karp et al., 2006). Specifically, leisure activities were categorized into cognitive activities and non-exercise physical activities (Zhu et al., 2017). Cognitive activities required a cognitive component participation, such as reading books, listening to the radio, playing cards and participation in organized activity.

Non-exercise physical activity was not intended to develop and maintain fitness, which included doing housework, outdoor activity, keeping domestic animals or pets and gardening. Therefore, the total scores for both cognitive activities and non- exercise physical activities ranged from 0 to 20. If the cognitive activity score was greater than the leisure activity score, the individual would be considered participating in a cognitive activity; otherwise, non- exercise physical activity. *T* test was performed to compare the score of cognitive ability between different types of leisure activities. And the result was *P* < 0.001, which indicated the significant difference between cognitive activities and non- exercise physical activities.

**FIGURE 8:**
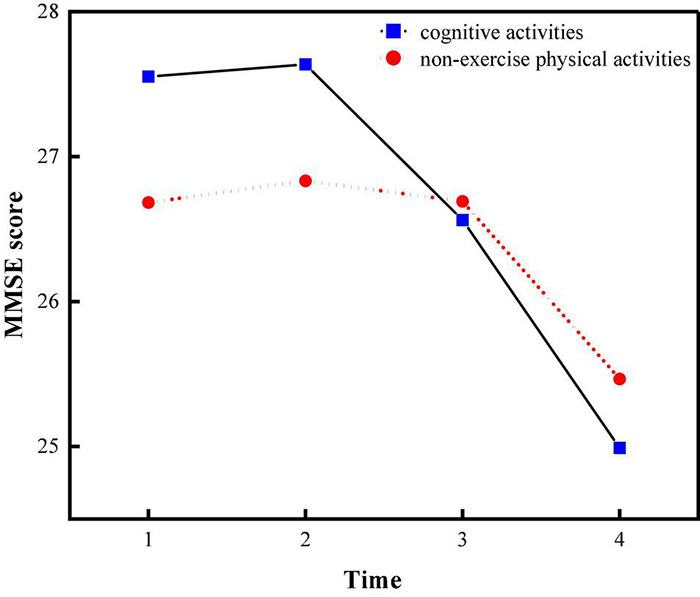
Cognitive ability scores for different types of leisure activities.

And the effect of different types of activities on cognitive ability was shown in Figure 8. It can be suggested from the Figure 8 that the cognitive ability of individuals who participated in cognitive activities was significantly higher than that of non-exercise physical activities participants in the first three periods. However, in the last period, it turned out the opposite.

Finally, it can be concluded that the impact of smoking on the cognitive ability was not significant in 2008, 2011, and 2018. In 2014, smoking had a significant impact on improving the cognitive ability of the older population (β = 0.537, SE = 0.236, *P* < 0.05). In addition, it was found that the effect of alcohol drinking on cognitive ability was positive in this study but it was not significant during the periods of four measurements.

### Parallel Development Model (Model 6)

**FIGURE 9:**
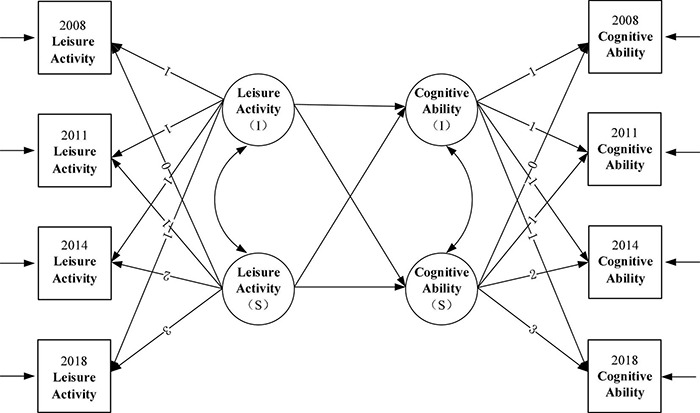
Parallel development model. I denote intercept; S denote slope.

In order to avoid measurement errors to more accurately examine the relationship between leisure activities and cognitive ability, a parallel growth model was developed to examine the influence process between leisure activities and cognitive ability by setting up a regression equation between growth factors, which was denoted as Model 6 (M6). The intercept and slope of leisure activities were used to predict the increase of cognitive ability. The conceptual model was shown in Figure 9.

The model fitting metrics were as follows: χ^2^(df) = 5.6, *P* = 0.000, CFI = 0.949, SRMR = 0.09; RMSEA = 0.042. In the latent variable parallel growth model of leisure activities and cognitive ability of older adults, the regression coefficient of the intercept of leisure activities of older people on the intercept of cognitive ability was significant (β = 0.43, *P* = 0.031), indicating that the higher the initial level of leisure activities, the higher the initial level of cognitive ability among the older adults. Hypothesis 4 was supported. The intercept of leisure activities of the older population influenced the slope of their cognitive ability (β = −0.40, *P* = 0.012), indicating that the higher the initial level of leisure activities, the slower the decline of cognitive ability. Hypothesis 5 was supported. The slope of leisure activities had no significant effect on the intercept of cognitive ability (β = 0.52, *P* = 0.09), suggesting that the initial level of cognitive ability would not be influenced by the change rate of leisure activities. The slope of leisure activities had a significant impact on the slope of cognitive ability (β = 0.82, *P* = 0.013), indicating that the faster the level of leisure activities of the older population decreased, the faster the level of cognitive ability decreased. Hypothesis 6 was supported. The specific fitting results of the model were shown in Table 5.

**TABLE 5:** Model fitting metrics.

Model	χ^2^(df)	CFI	SRMR	RMSEA	*P*
Model 5	3.2	0.929	0.055	0.046	0.000
Model 6	5.6	0.949	0.09	0.042	0.000

### Cross-Lagged Regression Analysis (Model 7)

**FIGURE 10:**
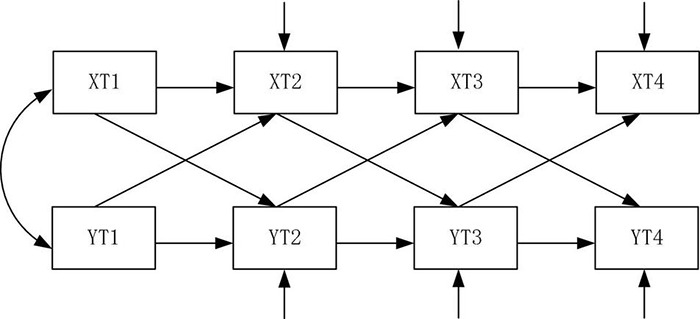
Cross-lagged regression model.

The latent variable growth model was used to study the dynamic characteristics of the variable. To further examine the leading lag relationship between leisure activities and cognitive abilities of the older population over time and to strengthen the demonstration for causal direction, the cross-lagged regression analysis was carried out in four measurements. Cross-lagged regression analysis can reveal complex relationships between two variables. The autoregressive effect of each variable was controlled by setting the stability coefficient, which was the best way to test the “Pure”effect among variables (Preacher, 2015) and used to understand how well one variable predicted another variable in general. A growing number of researchers believed that a combination of methods should be considered in order to obtain more robust conclusions in causal inference, thus allowing for sensitivity analysis in a broader sense (Curran and Bollen, 2001; De Stavola et al., 2006; Pakpahan et al., 2017). Therefore, the following cross-lagged regression model was constructed in this paper, as shown in Figure 10.

The results showed that the level of leisure activities of older adults in 2008 significantly positively predicted their cognitive ability in 2011 (β = 0.112, SE = 0.017, *P* = 0.000). The level of leisure activities of the older population in 2011 significantly positively predicted their cognitive ability in 2014 (β = 0.106, SE = 0.018, *P* = 0.000). The level of leisure activities of older adults in 2014 significantly positively predicted their cognitive ability in 2018 (β = 0.300, SE = 0.024,*P* = 0.000). Therefore, Hypothesis 7 was supported. Conversely, the level of cognitive ability of older adults in 2008 had no significant impact on their leisure activities in 2011 (β = 0.171, *SE* = 0.027, *P* = 0.061). The level of cognitive ability of older adults in 2011 had no significant impact on their leisure activities in 2014 (β = 0.188, SE = 0.026, *P* = 0.072). The level of cognitive ability of older adults in 2014 had no significant impact on their leisure activities in 2018 (β = 0.160, SE = 0.021, *P* = 0.085). The results also provided further evidence that there was no endogeneity problem in this study and the findings were scientifically valid.

## Discussion

To our knowledge, the current study was the first longitudinal study to investigate the change trajectory of leisure activity and long-term effects of leisure activity on cognitive ability. In addition, endogenous problems were considered in our study. What’s more, the study was based on a large, representative Chinese sample, which ensured the generalizability of the findings. Finally, both time-variant and time-invariant covariates on older population’s cognitive ability was assessed.

### Changing Trajectory of Cognitive Ability

According to the results of the unconditional linear model of the cognitive ability of the older population, the cognitive ability of older adults showed a significant downward trend from 2008 to 2018, which was consistent with the existing research conclusions (Konttinen et al., 2016). With the increase of age, the cognitive function of older people gradually weakened, and their general ability, reaction ability, attention and calculation ability, memory, language understanding and self-coordination ability also further declined, which were determined mainly by the decline of biological changes in the brain (Bäckman et al., 2000). In addition, Unconditional non-linear model indicated that the cognitive ability of the older people showed a trend of slow decline at the beginning and then accelerated decline, which was consistent with the theory of human cognitive aging (Wingfield and Grossman, 2006). Over time, cognitive function decline in the older population accelerated. Especially from 2014 to 2018, the cognitive ability of older adults showed a precipitous decline, which may be because during the four measurement periods, the older population gradually transferred from the young middle-aged elderly in 2008 to the middle-aged or older elderly in 2018. This was the period when the cognitive and physical functions of the older population degraded the most. Accordingly, the cognitive ability of older adults deteriorated drastically over time, especially in the late-life.

### Changing Trajectory of Leisure Activities

It was indicated that the level of leisure activities of older adults decreased significantly in the four measurement periods, which was consistent with previous studies (Feng et al., 2020). Since the subjects of this study were over 60 years old in the base period, with the increase of age, the older adults began to step into the recession period in the life cycle, and the physical function of older adults also decreased. Consequently, the elderly gradually suffered from diseases, such as arthritis, which severely restricted physical and recreational activities of the elderly (Zimmer et al., 1997). Even if leisure activities such as watching TV may increase over the late-life to some extent (Robinson et al., 2004), watching TV was a small part of all leisure activities which could not adverse the trajectory of leisure activities decline on the whole. In addition to changes in physical function, the reason for the decline in leisure activities among older adults may be due to changes in today’s social interaction patterns. Nowadays, it was not uncommon for older adults to be unfamiliar with their next-door neighbors and thus social interactions-rated activities decreased (Feng et al., 2020). Furthermore, unconditional non-linear model indicated that the leisure activities of the older people showed a trend of slow decline at the beginning and then accelerated decline, which was line up with the development of physical function (Xu et al., 2018). After stepping into the oldest-old stage, it was uncommon and hard for older adults to maintain regular leisure activities. As a result, leisure activity levels in older adults declined at an accelerated rate later in the life cycle. Accordingly, the overall level of leisure activities of the older population gradually showed a downward trend over time.

### Effects of Time-Variant and Time-Invariant Covariates on Cognitive Ability

Through the study of model 5, it was found that the cognitive ability was influenced by the level of education. Specifically, the higher the level of education, the higher the cognitive ability at the same period, which could be explained by that the older people with high education level are more likely to engage in work related to cognitive tasks, such as reasoning and memory and their cognitive ability will be improved through processing these tasks (Lindenberger et al., 1993). In addition, it was found that the higher level of education, the slower decline of cognitive ability, which was in line with the previous study and could be explained by that the likelihood of cognitive ability decline associated with lacunar infarcts was lower among individuals with high education compared to those with low education (Farfel et al., 2013). Education was a kind of solidified intelligence obtained at a young age, which can maintain a better condition for older adults and slower the degradation of cognitive ability (Mazzonna and Peracchi, 2012).

In addition, Although most studies indicated that smoking can significantly reduce cognitive ability (Ott et al., 2004; Nooyens et al., 2008), the positive effects of smoking on the cognitive ability of the older adults was found in 2014, which may be explained by the neuroprotective effects of nicotine in cigarettes from a biological point of view (Kihara et al., 1998; Mihailescu and Drucker-Colín, 2000). Additionally, the frequency of smoking was the key factor affecting cognitive ability. Due to the smoking habits and social habits of the older population, the smoking frequency of older adults was generally lower than that of the young. Therefore, it was found that smoking can improve the cognitive ability of the older population to a certain extent.

Similarly, it was revealed that alcohol drinking was a risk factor for the cognitive ability of the older population (Katja et al., 2014), however, it was found that the effect of alcohol drinking on cognitive ability was protective in this study even if it was not significant, which may be due to differences in the amount of alcohol drinking. A small amount of alcohol drinking was beneficial to physical and mental health, while excessive drinking will damage brain cells, thus damaging the cognitive function of the older adults. In addition, it may be related to the frequency of alcohol drinking. Regular and small consumption was beneficial to cognitive function, while occasional drinking will reduce the cognitive ability of older people (Horvat et al., 2015; Reas et al., 2016).

Last but not least, the positive impact of leisure activities on the cognitive ability of the older population was suggested. Specifically, it was indicated that the level of leisure activities of older adults significantly positively predicted their cognitive ability during the same period. In other words, the higher the level of leisure activities in 2008, the higher the level of cognitive ability in 2008. Similarly, the positive prediction relationship was still significant in 2011, 2014 and 2018, which can be explained by the cognitive reserve theory that the older population’s participation in leisure activities may produce a more effective cognitive network, so as to provide a cognitive reserve and delay the decline of their physiological cognitive ability (Scarmeas and Stern, 2003; Cristina et al., 2010).

In addition, from the perspective of different types of activities, cognitive activities had a greater effect on cognitive ability than non- exercise physical activities during the first three measurement periods, Cognitive activities maintained the cognitive ability of older adults by stimulating brain activity. Therefore, during the first three measurement periods, the cognitive ability of the older population who participated in cognitive activities was significantly higher than the counterpart who participated in non- exercise physical activities.

### Influence Process of Leisure Activities on the Change of Cognitive Ability

It was suggested that initial level of leisure activities in 2008 not only predicted the initial level of cognitive ability in 2008 but also the level of cognitive ability in 2011, 2014, and 2018. In addition, through the parallel development model, it was revealed that the higher the initial level of leisure activities, the slower the decline of cognitive ability, which was consistent with the previous analysis, that was, leisure activities were the protective factor of the cognitive ability of older adults (Park et al., 2019). The higher the level of leisure activities of the older population can effectively inhibit the decline of their cognitive ability. Cause it has been suggested that participation in leisure activities may provide cognitive reserve as an attitude toward an active lifestyle in older adults, which may delay the clinical manifestations of cognitive decline (Cristina et al., 2010). Furthermore, it was demonstrated that the rate of decline in leisure activities of older adults can predict the rate of decline in their cognitive ability, which meant that the faster the level of leisure activities decreased, the faster the level of cognitive ability decreased. On the contrary, the cognitive decline was also slower among older adults whose leisure activity levels declined more slowly, which indicated that the level of leisure activities and the level of cognitive ability had the consistent change trend, so the slope of leisure activities was significantly related to the slope of cognitive ability, and the change direction was consistent.

Finally, a cross-lagged regression analysis was conducted on the older population’s leisure activities and cognitive ability, in order to explore the time sequence between leisure activities and cognitive ability. As a result, it was examined that the level of leisure activities of the older population could positively predict the subsequent cognitive ability, which further verified the causal inference between the current leisure activities and cognitive ability among older adults.

## Study Limitations and Recommendations for Future Research

The study was subject to a few limitations. First, this paper only considered the influence of educational factors on cognitive ability, and other time-invariant factors, such as gender, marriage, and region, had not been considered. Therefore, future studies should take more demographic variables into consideration. In addition, Since CLHLS was a face-to-face questionnaire, the older population’s answers to leisure activity-related questions were only a subjective perception. Furthermore, the measurements of leisure activities were based on frequency in this study, but the quality and duration of leisure activity was more important. Therefore, in the future, objective data and the quality of leisure activities could be better to measure the leisure activities of the older population.

## Conclusion

Based on the above discussion and analysis, the following research conclusions were drawn from this paper:

(1) On the whole, the level of cognitive ability of older adults showed a non-linear decreasing trend, and the decreasing trend gradually increased over time. In addition, there was significant difference in the initial level and the rate of change of cognitive ability among older adults.
(2) Overall, the level of leisure activities of the older population showed a non-linear decreasing trend, and the decreasing trend gradually increased over time. What’s more, there were differences in the initial level of leisure activity and no differences in the decline rate of leisure activity level among the older population.
(3) At every time point, the level of leisure activities had a significant positive impact on cognitive ability among older people, that was, the higher the level of leisure activities, the higher the level of cognitive ability. In addition, cognitive activities had a greater effect on cognitive ability than non- exercise physical activities.
(4) The older population with high level of initial leisure activities had higher initial level of cognitive ability, and the decline of cognitive ability was slow.
(5) The faster the level of leisure activities decreased, the faster the level of cognitive ability decreased.

(6) The level of leisure activities in the previous period can positively predict the cognitive ability of the older population in the later period.
(7) Education can significantly promote the initial cognitive ability of older adults, and the higher the level of education, the slower the decline of cognitive ability.
(8) For the older population, smoking shed a significant positive effect on cognitive ability to some extent and no significant effect was found between alcohol drinking and cognitive ability.

Overall, the correlation between cognitive ability and leisure activity suggested that more targeted interventions should be undertaken to promote existing leisure activities among older adults, especially cognitive activities.

## Data Availability Statement

The datasets presented in this study can be found in online repositories. The names of the repository/repositories and accession number(s) can be found in the article/Supplementary Material.

## Ethics Statement

The studies involving human participants were reviewed and approved by the Ethics Committee of Peking University (IRB00001052–13074). Written informed consent for participation was not required for this study in accordance with the national legislation and the institutional requirements.

## Author Contributions

CZ: data curation, formal analysis, methodology, and resources. XZ and LZ: funding acquisition. All authors contributed to the article and approved the submitted version.

## Conflict of Interest

The authors declare that the research was conducted in the absence of any commercial or financial relationships that could be construed as a potential conflict of interest.

## Publisher’s Note

All claims expressed in this article are solely those of the authors and do not necessarily represent those of their affiliated organizations, or those of the publisher, the editors and the reviewers. Any product that may be evaluated in this article, or claim that may be made by its manufacturer, is not guaranteed or endorsed by the publisher.
